# The Expansion of *Dirofilaria repens* in the Irtysh Basin of Western Siberia Is Associated with Nine Species of *Aedes* Mosquitoes

**DOI:** 10.3390/insects17040398

**Published:** 2026-04-07

**Authors:** Vladimir A. Burlak, Tatyana A. Khlyzova, Valentina S. Fedorova, Yuliya V. Andreeva, Svetlana S. Alekseeva, Dmitry A. Karagodin, Igor V. Sharakhov, Maria V. Sharakhova, Gleb N. Artemov

**Affiliations:** 1 Department of Genetics and Cell Biology, Tomsk State University, Tomsk 634050, Russia; flywings@mail.ru (V.A.B.); igor@vt.edu (I.V.S.); g-artemov@mail.ru (G.N.A.); 2 Tobolsk Complex Scientific Station, Ural Branch of the Russian Academy of Sciences, Tobolsk 626152, Russia; khlyzovata@tobscience.ru; 3 Laboratory of Biology and Soil Science, Tomsk State University, Tomsk 634050, Russia; 4 Laboratory of Cell Differentiation Mechanisms, Institute of Cytology and Genetics, Novosibirsk 630090, Russia; karagodin@bionet.nsc.ru; 5 Department of Entomology, Virginia Polytechnic Institute and State University, Blacksburg, VA 24061, USA; 6 Fralin Life Sciences Institute, Virginia Polytechnic Institute and State University, Blacksburg, VA 24061, USA

**Keywords:** *Dirofilaria* spp., *Dirofilaria repens*, mosquitoes, Culicidae, *Aedes*, infection transmission, vector species, xenomonitoring, helminth-host interaction

## Abstract

The northward expansion of *Dirofilaria* spp. is a current medical and veterinary concern. However, it is unclear how far north the parasite has spread in Western Siberia and what species of mosquito can carry and transmit it. This study examined *Dirofilaria* spp. infection in *Aedes* mosquitoes in the taiga zone of the Irtysh Basin. The mosquito species were identified based on morphology, and *Dirofilaria* spp. were identified using PCR. Of the 13 habitats surveyed, 24 of 2205 mosquito samples were infected with *D. repens* and 1 with *Dirofilaria* sp. The highest infection rate (~7.6) was recorded in Tobolsk, Tyumen region, at 58.4° N. Mosquito infection was recorded as far north as 61° N in Khanty-Mansiysk. The presence of the infective L3 stage of the parasite was recorded up to 60° N in Bobrovsky. Nine species of mosquitoes were found to be infected with *D. repens*: *Aedes rossicus*, *Aedes behningi*, *Aedes cantans*, *Aedes communis*, *Aedes cyprius*, *Aedes euedes*, *Aedes excrucians*, *Aedes flavescens*, and *Aedes sticticus*. Two of these species, *Ae. behningi* and *Ae. communis*, were competent vectors of the parasite. Thus, *D. repens* has successfully adapted to the *Aedes* mosquito in the taiga zone of Siberia.

## Simple Summary

Dirofilariasis, a disease caused by an invasive zoonotic nematode, has spread significantly over the last three decades due to climate warming and human movement. In Eurasia, the parasite has rapidly moved north and east, successfully adapting to local conditions, and Russia stands out as a country where dirofilariasis is spreading particularly quickly. However, the forces that enable the parasite to adapt to new mosquito vectors and external incubation conditions, as well as biotic and abiotic factors in native habitats, are poorly understood. This study showed that *Dirofilaria repens*, the agent of subcutaneous and ocular dirofilariasis, has adapted as far north as 61° N in the taiga zone of the Irtysh and Ob River Basins. This study identified the mosquito species associated with *D. repens* infections and the species that are competent vectors of the disease. Our study also determined biotic and abiotic factors influencing the parasite’s spread. We found that mosquito species infected with *D. repens* were also infected with local helminths, which may enhance their ability to transmit *Dirofilaria* spp. Overall, this information is important for understanding the parasite’s adaptation strategies in the taiga zone of Western Siberia and for developing effective disease-fighting strategies.

## 1. Introduction

Dirofilariasis is a zoonotic disease of growing medical and veterinary importance in the 21st century [1]. The disease is primarily associated with the nematode species *Dirofilaria immitis* (Leidy, 1856) and *Dirofilaria repens* (Railliet & Henry, 1911), belonging to the order Spirurida and the family Onchocercidae. These species are heteroxenous helminths whose definitive hosts are domestic dogs and cats, as well as other carnivores and humans. *D. immitis*, the dog heartworm, causes pulmonary disease in humans, while *D. repens* causes subcutaneous infections under the skin or in the eye. In Europe, the intermediate hosts for *D. repens* and *D. immitis* are mosquitoes of the *Anopheles* genus, particularly *An. maculipennis* s.l., along with *Culex pipiens*, *Aedes vexans*, *Ae. caspius*, and *Ae. albopictus*, as well as several secondary vectors [1]. Upon entering a mosquito, the microfilaria undergoes metamorphosis in the Malpighian tubule and molts twice, maturing into an infective L3 stage larva. It then migrates to the female mosquito’s salivary glands, where it becomes infective and enters the proboscis. The infective larva enters the definitive host during mosquito blood-feeding on a vertebrate animal, where it molts three more times and continues to mature for approximately nine months until reaching the adult stage. Recently, dirofilariasis has spread from endemic zones, such as the Mediterranean, in northward and eastward directions across Eurasia [1]. The number of human cases is increasing in Europe and Russia, doubling every 3 years from 1996 to 2012 [2]. With each summer, the geographic range of human and canine cases has expanded, reaching as far as the 60th parallel in the European part of Russia and Finland [3,4]. Mosquito infections in Western Siberia reach the 63rd parallel [5], and dog infections in Yakutia (Sakha), Northeast Asia, reach the 62nd parallel [6]. Global warming, increased human and animal movement, and other human activities are worsening the situation and promoting the further spread of the disease [1,7]. Therefore, the current spread of dirofilariasis is an emerging yet neglected problem that requires further evaluation [8].

Although the medical and veterinary aspects of dirofilariasis have been thoroughly studied, the relationship between *Dirofilaria* spp. and intermediate mosquito hosts at the population and species levels remains poorly understood [8]. This is especially true in the areas recently affected by the parasite’s expansion. The lack of research on this topic stems from the parasite’s widespread expansion and the difficulty of identifying mosquito species [9,10,11]. Another reason is the morphological similarity between the larvae of *D. repens* and *D. immitis*, both of which parasitize the Malpighian tubules of mosquitoes [12]. In recent years, molecular diagnostic technologies have become valuable tools for identifying parasite and host species, providing new insights into the relationship between vectors and parasites [13]. However, such studies in Russia began only in the last decade [5,14,15,16,17,18,19,20]. Understanding the geographic expansion of *Dirofilaria* spp., its relationship with specific vector species, and its life cycle—including the development of the third-stage larva (L3), which is infectious to vertebrate hosts—within the vector species can help to better understand how parasite populations adapt to local conditions and the danger they pose to definitive hosts. Climate warming also complicates the situation by altering the ecology of vectors at the organizational and population levels, as well as their geographical distribution in many countries [1]. Therefore, data from earlier studies, conducted before the development of molecular techniques, must be revisited and updated.

In Russia, autochthonous dirofilariasis has been recorded in the Altai region of Western Siberia since the late 1980s [21]. Within ten years, the parasite spread northwestward to Kurgan, Omsk, and Novosibirsk. Over the next 10 to 15 years, it spread northeastward to the Khanty-Mansiysk Autonomous Region, the Tomsk Region, and the Krasnoyarsk Territory [22,23,24,25,26,27]. Currently, *Dirofilaria* spp. are expanding their geographic range northward due to their successful adaptation to local vector populations in previously unknown ecosystems [1,5,13,18,20,28]. For example, *D. immitis* was detected in intermediate hosts up to 58.5° N around Kolpashevo and *D. repens* up to 62.5° N in the Priobye village of the Khanty-Mansiysk Autonomous Okrug [5,20]. Prior to this study, xenomonitoring in the Upper and Middle Ob basin identified 19 (57.6%) of 33 mosquito species as positive for *Dirofilaria* spp.; however, only 4 were proven vectors: *Anopheles beklemishevi*, *Anopheles daciae*, *Anopheles messeae*, and *Aedes cantans* [5,18,19,20]. However, the spread of *Dirofilaria* spp. in the Kurgan and Tyumen regions, the Khanty-Mansi Autonomous Okrug, and the Yamalo-Nenets Autonomous District warrants further attention, as this area serves as a natural intermediary between the Urals and the Ob region.

Historically, dirofilariasis has manifested as isolated cases of human disease, then remained dormant for about a decade. Then, it reappears with a significantly larger number of human infections. This pattern reflects the parasite’s adaptation to local conditions, including vector species and populations, as well as to the seasonal accumulation of effective temperatures over time, against the backdrop of ongoing climate warming [1,13,29,30]. The relationship between *Dirofilaria* spp. larvae and their intermediate hosts is closely linked to their adaptation to local abiotic factors, such as temperature and humidity, as well as biotic factors, including native mosquito species, their population structures, and symbiotic microbiota [5,28,29,31,32]. However, processes of helminth transmission, communities of vector species across different climatic zones, and their vector competence have been poorly studied. Thus, a comprehensive study of *Dirofilaria* spp. adaptation in taiga zone mosquito populations is necessary to understand the effectiveness of parasite adaptation strategies.

**Figure 1:**
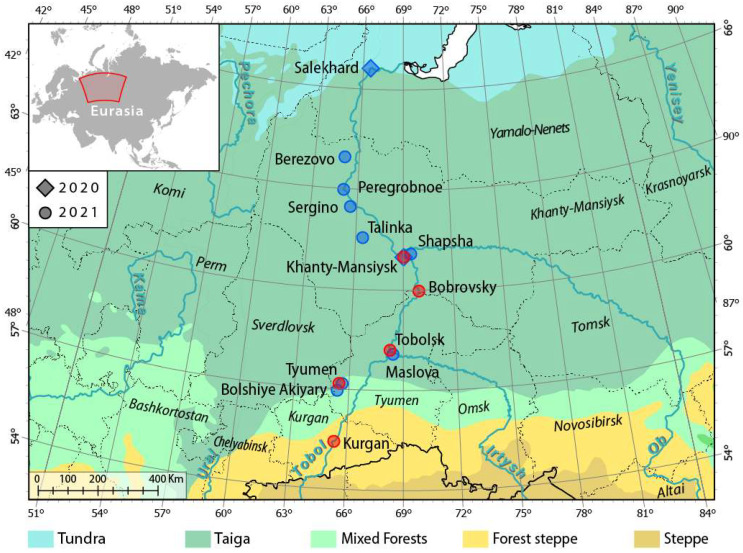
Mosquito collection sites in the Irtysh Basin of Western Siberia. The location of the examined region is shown in the corner of the Eurasia map. Red and blue dots indicate locations where *Dirofilaria repens* infections were and were not found, respectively. The ecoregions are shown in different colors. Administrative divisions are shown by dashed lines. The names of administrative regions are shown in italics.

For the first time, molecular polymerase chain reaction (PCR) diagnostics were used in this study to identify *Dirofilaria* spp. in a large pool of *Aedes* mosquitoes collected in the Irtysh region, focusing specifically on the area between Kurgan and Khanty-Mansiysk (Figure 1). The goals of our study are as follows: (1) to identify *Aedes* species associated with *Dirofilaria* spp. in the taiga zone of the Irtysh Basin and the Ob River below the Irtysh confluence, (2) to determine the species that are competent vectors of *Dirofilaria* spp. in this region, (3) to delineate the current geographic spread of *Dirofilaria* spp., and (4) to identify biotic and abiotic factors influencing the parasite’s spread. In this study, we focused specifically on *Aedes* mosquitoes because the entomological net used to collect them mostly captured species from the genus *Aedes*, not from other mosquito genera. This information helped us better understand the parasite’s adaptation strategies in Western Siberia and provided additional details on the composition of mosquito species in this region and their relationships with *Dirofilaria* spp. and related helminths.

## 2. Materials and Methods

### 2.1. Mosquito Collection and Identification

**Figure 2:**
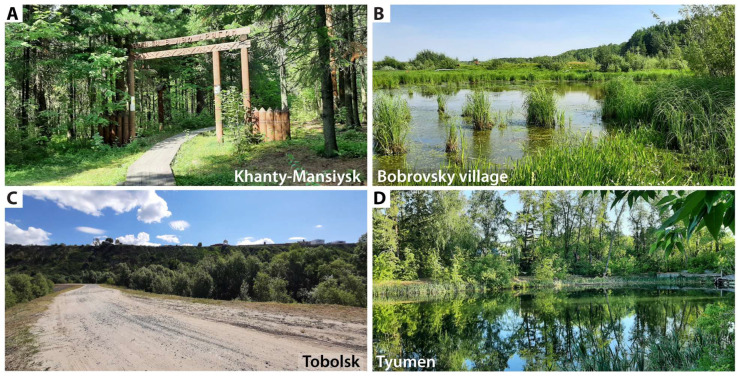
Collection sites where mosquitoes were infected with *Dirofilaria repens*. (**A**) Samarovsky Chugas A park, Khanty-Mansiysk, 3 August 2020. (**B**) Bobrovsky, floodplain of the Bobrovka River, 2 August 2020. (**C**) Tobolsk, Irtysh floodplain, 25 June 2021. (**D**) Tyumen, Olovyannikov pond, 24 June 2021.

Mosquitoes were collected along the Irtysh and Ob Rivers and their tributaries in the West Siberian Plain. The geographical scope of the study, the locations of the sites, and the timing of the collections are shown in Table 1 and Figure 1. The mosquitoes were captured using an entomological net, as this was the most cost-effective method, and then immobilized by freezing them briefly at −20 °C on the day of collection. Descriptions of the biotopes are provided in Table S1; photographs of the collection sites are shown in Figure 2 and Figure S1. In remote areas such as Salekhard, Berezovo, Khanty-Mansiysk, and Tobolsk, where it was not possible to perform species composition analysis in the laboratory on the day of collection, the mosquitoes were frozen or fixed in nicotine. They were then laid out on cotton mats, dried, and stored at room temperature for later identification. The species composition was first determined using dichotomous keys [33]. After identification, the mosquitoes were fixed in 96% ethanol and stored at −20 °C. Morphological identification failed for four infected specimens from Bobrovsky and Tobolsk. Instead, the mitochondrial cytochrome oxidase subunit I (COI) gene was sequenced. Then, the sequences were compared with those in the GenBank database using BLAST 2.17.0 to determine their similarity (https://www.ncbi.nlm.nih.gov/genbank/, accessed 31 December 2025). In addition, we identified the species of malaria mosquitoes using the length of the internal transcribed spacer 2 (ITS2) of the ribosomal DNA as a marker with PCR and (restriction fragment length polymorphism) RFLP assays [34].

### 2.2. Dirofilaria spp. Detection and Identification

To detect *Dirofilaria* spp. larvae and other parasites, we dissected female adult mosquitoes using a previously described method [20] under an MBS-12 stereomicroscope (LZOS, Moscow, Russia) in a drop of PBS solution (137 mM NaCl, 10 mM Na_2_HPO_4_, 2.7 mM KCl, and 1.8 mM KH_2_PO_4_). The dissected parts of the mosquito body in PBC solution, such as Malpighian tubules, stomach, esophagus, posterior intestine, ovaries, salivary glands, and proboscis, were covered with a coverslip and examined under an AxioStar microscope (Carl Zeiss, Göttingen, Germany) at 60×, 150×, and 600× magnifications. All infection variants (helminthiasis, protozoosis, mycosis, and hydrachnidiosis) were recorded using a digital camera (Smartphone Camera, Galaxy A50, Samsung, Suwon, Republic of Korea). After detecting *Dirofilaria* spp. infection, the mosquitoes were individually washed and fixed in 96% ethanol in 0.5 mL Eppendorf tubes.

The samples that tested positive for *Dirofilaria* spp. by microscopic analysis were then analyzed by PCR. DNA extraction was performed from the fixed material, which contained both mosquito and parasite genomic DNA. This was performed following the previously described protocol [35] with modifications. The material was removed from the fixative solution, dried at room temperature, and homogenized in 50 µL of STE buffer solution (100 mM NaCl, 10 mM Tris-HCl [pH 8.0], and 1 mM EDTA). The homogenate was incubated at 95 °C for five minutes and then immediately frozen at −20 °C. After that, it was completely thawed to release DNA from the tissues into the solution through subsequent centrifugation at 10,000 g for five minutes. The DNA-containing supernatant was transferred into 1.5 mL microcentrifuge tubes and stored at −20 °C until used in the PCR. A similar DNA extraction technique was used to analyze whole dried mosquitoes. To identify *D. repens* and *D. immitis*, primers were based on differences in the nucleotide sequence of the mitochondrial cytochrome oxidase I (COI) gene [36]. The primers DR COI-F1 (5′-AGTGTTGATGGTCAACCTGAATTA-3′) and DR COI-R1 (5′-GCCAAAACAGGAACAGATAAAACT-3′) were used to identify *D. repens*, and the primers DI COI-F1 (5′-AGTGTAGAGGGTCAGCCTGAGTTA-3′) and DI COI-R1 (5′-ACAGGCACTGACAATACCAAT-3′) were used to identify *D. immitis*. The 20 µL reaction mixture contained either 10 pmol of DR COI-F1 and DR COI-R1 or DI COI-F1 and DI COI-R1; 1× dNTP, 1× PCR buffer, and 0.5 units of Taq polymerase (Biolabmix, Novosibirsk, Russia); and approximately 0.1 µg of total DNA. PCR was performed in 32 cycles according to the following protocol: denaturation at 94 °C for 30 s, annealing at 57 °C for 30 s, and elongation at 72 °C for 30 s. Initial denaturation was performed at 95 °C for five minutes, and terminal elongation was performed at 72 °C for seven minutes.

To determine infection in whole dried mosquitoes, PCR was first performed using universal primers for the internal transcribed spacer (ITS2) DIDR-F1 (5′-AGTGCGAATTGCAGACGCATTGAG-3′) and DIDR-R1 (5′-AGCGGGTAATCACGACTGAGTTGA-3′) to determine filariasis. The reaction mixture contained the same reagent composition as the PCR mixture for COI analysis, except for the primers. PCR was performed using the same protocol. The length of the PCR product was used to determine the filarial genus and *Dirofilaria* spp. [36]. To clarify the results, samples that tested positive (presence of a PCR product of approximately 500 bp) with the universal primers were reanalyzed with COI-specific primers, as described previously. To determine the *Dirofilaria* spp. in dried female mosquitoes, individual analyses (Salekhard on 9 August 2020, and Tobolsk on 18 September 2021) and pools of five specimens were used (Khanty-Mansiysk and Berezovo). The extent of the infection was calculated as a percentage, representing the ratio of infected individuals to the total number of individuals analyzed. For samples in which mosquitoes were pooled for analysis, the minimum infection rate (MIR), defined as the ratio of pools with detected infection to the total number of pools, was calculated [17].

### 2.3. Calculation of Transmission Cycle (TC) Number

Data on local habitat temperatures were obtained from the Weather and Climate website archives (http://www.pogodaiklimat.ru, accessed 31 December 2025). The number of transmission cycles (TC) or number of parasite development cycles to the infective L3 stage was calculated using the following formula: number of TC = SET/130, where TC is the number of transmission cycles; SET is the sum of effective temperatures on the sampling day or over the season, defined as ∑(T °C daily mean − T °C threshold), with the threshold temperature set at 14 °C; and 130 is the total heat requirement in degree-days (DD) above 14 °C, which is needed for one complete parasite development cycle [37]. The number and timing of TC, as well as the duration of the daily infection cohort (mosquitoes infected on the same day and developing in parallel), were calculated using the extended daily mean [29] or hourly model [38].

## 3. Results

**Figure 3:**
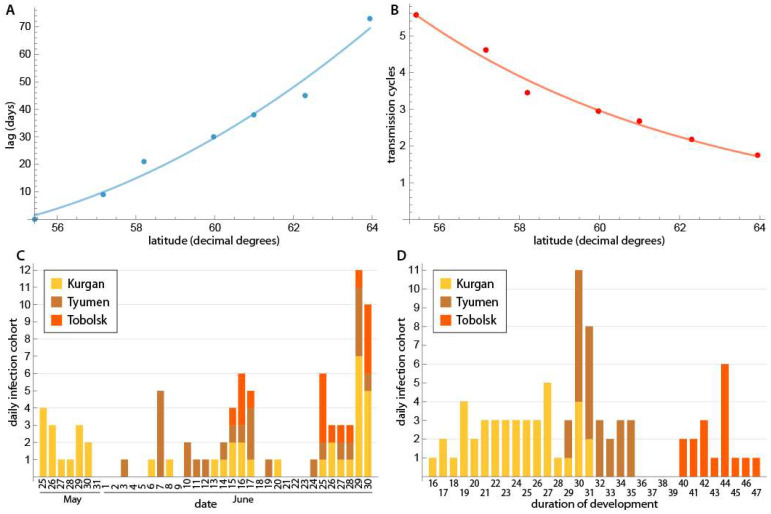
The parameters of *D. repens* development at mosquito collection sites. (**A**) The order of maturation of the first transmission cycle (TC) in the Ob–Irtysh Basin habitats in the 2021 season is as follows: Kurgan (25 May) is assumed to be zero, followed by Tyumen (3 June), Tobolsk (15 June), Bobrovsky (24 June), Khanty-Mansiysk (2 July), Priobye (Oktyabrskoye, 9 July), and Berezovo (7 August). The average delay per degree of latitude is 8.7 days. (**B**) The relationship between the number of TC per season and the latitude of the Ob–Irtysh Basin habitats (average daily model): Kurgan: 5.57; Tyumen: 4.61; Tobolsk: 3.45; Bobrovsky: 2.95; Khanty-Mansiysk: 2.68; Ob region: 2.18; Berezovo: 1.75. On average, the number of TC decreases by 0.45 per latitudinal degree. (**C**) The number of maturation days of daily infection cohort development in three Irtysh Basin habitats (25 May–30 June 2021; average daily model): Kurgan: 20 days of maturation in 37 calendar days (54%), with 41 daily infection cohorts. Tyumen: 16 days of maturation in 27 calendar days (59%), with 26 mature daily infection cohorts. Tobolsk: 9 days of maturation in 16 calendar days (56%), with 17 mature daily infection cohorts. (**D**) The duration of development (in days) and the number of mature daily infection cohorts in the three Irtysh Basin habitats (25 May–30 June 2021; average daily model) at the time of the study. In Kurgan, the duration of the 476 daily infection cohorts ranged from 16 to 31 days; in Tyumen, from 29 to 35 days; and in Tobolsk, from 40 to 47 days. The duration of the parasite’s development is determined by external incubation temperatures (the sum of effective temperatures).

This study examined *D. repens* infections in *Aedes* mosquitoes collected along the Irtysh and Ob Rivers and their tributaries in the West Siberian Plain (Figure 1, Table 1). A total of 2205 females from 17 collection sites in 13 habitats were collected and analyzed from 7 August 2020 to 18 September 2021 (Figure 1, Table 1). Within the longitudinal corridor of 65–70° east, the northern collection point (Salekhard neighborhood, 66°37′23″ N) and the southern collection point (Kurgan center, 55°25′38″ N) were slightly over 11 latitudinal degrees apart, or about the Euclidean distance of 1234 km from each other, and the driving distance was 2295 km. To assess the potential for parasite development, we calculated external incubation temperatures (EITs) in the habitats. To better understand the parasite development stage, we estimated the number of transmission cycles (TCs), as described in the Materials and Methods section. Figure 3 summarizes the parameters of potential *D. repens* development in different collection sites. In 2020, the TC was 0.8 in Salekhard and 2.88 in Khanty-Mansiysk. The TC decreased inversely proportionally to latitude in 2021, ranging from 5.57 in Kurgan to 1.75 in Berezovo. On average, the change in TC was 0.45 per degree of latitude. The maturation date of the first TC increased with latitude. The average change step was 8.7 days per degree of latitude (Figure 3A,B). In Kurgan, the development of the first TC ended on 25 May, and the development of the second TC ended on 25 June. The duration of each daily infection cohort (mosquitoes infected on the same day and developing in parallel) from 25 May to 30 June (n = 41) was 16–31 days, averaging 23.9 days. In Tyumen, the first TC matured on 3 June, and the second TC matured on 1 July. The duration of daily infection cohort development ranged from 29 to 35 days (n = 26), averaging 31.7 days. In Tobolsk, the first TC matured on 15 June and the second TC on 20 July. The duration of daily infection cohort development ranged from 40 to 47 days (n = 17), with an average of 41.3 days (Figure 3C,D). The development of the first TC in Bobrovsky was completed by 26 June (development duration: 50–52 days), while the development in Khanty-Mansiysk was completed by 3 July (development duration: 54 days). On average, the duration of daily infection-cohort development increased by 5 days per latitude degree.

In 17 collections, 28 species of blood-sucking mosquitoes were identified, including 21 species of genus *Aedes*, 3 species of genus *Culiseta*, 2 species of genus *Anopheles*, and 1 each of the genera *Culex* and *Coquillettidia*, but another 12.4% of individuals could not be identified by morphological characteristics (Table S2). Due to the significant latitudinal shift (about nine degrees) between Kurgan and Berezovo, mosquito populations in June were in different maturation phases. In the south, summer-drought-tolerant forest-steppe species dominated; in the north, spring and early-spring taiga species dominated. Most of the samples were collected during the last ten days of June, when daylight hours are at their maximum, and the number of mosquitoes reaches its peak [39]. Representatives of the genus *Anopheles* were not specifically studied in this work; however, 14 samples were found in two biotopes: three females of *An. messeae* Falleroni, 1826; five females of *An. daciae* Linton, Nicolescu et Harbach, 2004; six males of both species (not listed in the tables) in Tyumen, the 23 June 2021, 3 p.m. sample; and one female of *An. messeae* s.l. in Maslova.

**Figure 4:**
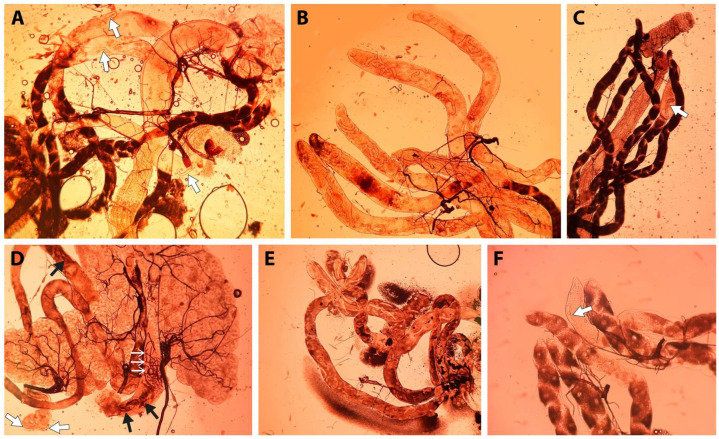
Examples of *Dirofilaria repens* infection found in dissected adult mosquitoes. (**A**) Female *Ae. cyprius* infected with *D. repens*, Kurgan, 22 June 2021. Larvae L1 are indicated by the white arrows. (**B**) Female *Ae. euedes* infected with *D. repens*. Tyumen, Olovyanikov Pond, 24 June 2021. (**C**) Female *Ae. excrucians* with a single *Dirofilaria sp.* infection. Tyumen, Olovyanikov Pond, 24 June 2021. Larva L2 is shown by wide white arrow. (**D**) Female *Ae. euedes* infected with *D. repens*. Most of the *D. repens* larvae are melanized at the microfilariae stage and shortly after metamorphosis, Tobolsk, 25 June 2021. White thin arrows show melanized microfilaria. Black wide arrows show melanized L1 larvae. Live larva L1 is shown by wide white arrow. (**E**) Female *Ae. cantans* infected with *D. repens*, Tobolsk, 21 June 2021. (**F**) Female *Ae. rossicus* with a single *D. repens* infection, Tobolsk, 21 June 2021. Larva L2 is shown by wide white arrow.

A total of 2205 female mosquitoes were included in the analysis. Among them, 1736 females were dissected and identified by their morphological characteristics [33], 20 of which were found to be infected with *D. repens* larvae (Figure 4A–F). Four of the infected females could not be identified based on morphology alone, so COI gene sequencing was used to identify them [36]. Two females from Bobrovsky were identified as *Ae. communis* (De Geer, 1776), showing 100% identity. Of the two unidentified infected females in Tobolsk, one was identified as *Ae. sticticus* (Meigen, 1838), and the other was assigned to the Annulipes Group due to the insufficient sequence diversity of the COI gene in GenBank (https://www.ncbi.nlm.nih.gov/genbank/, accessed 31 December 2025). The remaining 469 individuals were divided into multiple pools of 1–5 individual mosquitoes associated with different mosquito species. These pools of dried samples were examined for the presence of nematodes using PCR analysis [36]. A total of 25 cases of *D. repens* infections were detected. Among the specimens tested microscopically, 23 were identified as *D. repens*. One larva in the first half of the L2 stage from Tyumen was identified as *Dirofilaria* sp. in a female *Ae. excrucians* (Walker, 1856) by microscopic analysis (Figure 4C). Of all the samples identified using PCR on pool samples, only one from Khanty-Mansiysk tested positive for *D. repens* DNA on 28 June 2021. In addition to *D. repens*, analysis of a Salekhard sample on 9 August 2020 revealed an infection with the nematode *Setaria* sp. in a female *Ae. riparius* (Dyar & Knab, 1907).

*Dirofilaria repens* larvae were found in five habitats in the zone from 55°25′ N to 61°03′ N: Kurgan, Tyumen, Tobolsk, Bobrovsky, and Khanty-Mansiysk (Table 1 and Table S1). Most of the infections (91.7%) occurred in urban habitats (Kurgan, Tyumen, Tobolsk, and Khanty-Mansiysk), which can be defined as suburban forest parks of natural or artificial origin. The remaining infections (8.3%) occurred in rural marginal biotopes and mixed floodplain forests with abundant waterlogging (Bobrovsky). Tobolsk had the highest extent of infection (EI = 7.6 ± 1.8%), which differed significantly from the EIs of Tyumen and Kurgan (*p* < 0.01 in both cases). In southern habitats, the weather did not favor microfilariae development due to atypical heat and drought, so the EI of Tyumen and Kurgan populations did not differ: 0.8% ± 0.5% in Kurgan and 1.4% ± 1.0% in Tyumen (Table 1). Vector-competent females ready to transmit infection (with L3 *D. repens* larvae in their proboscises) were recorded in two locations: Tobolsk (*Ae. behningi*, Martini, 1926; development time: 35 days) and Bobrovsky (*Ae. communis*; development time: 50–52 days). As of 28 June 2021, the EI in Khanty-Mansiysk was 0.4% ± 0.4%. This is the first report on the vector properties of these species in Western Siberia.

The results of the analysis of mosquito species infected with *D. repens* larvae in Khanty-Mansiysk, Bobrovsky, Tobolsk, Tyumen, and Kurgan revealed nine species: *Ae. rossicus* (Dolbeshkin, Gorickaja, & Mitrofanova, 1930); *Ae. behningi; Ae. cantans*, Meigen, 1818; *Ae. communis*; *Ae. cyprius*, Ludlow, 1920; *Ae. euedes*, Howard, Dyar, & Knab, 1913; *Ae. excrucians*; *Ae. flavescens*, Müller, 1764; and *Ae. sticticus.* These species accounted for 34.6% of the total diversity identified. In Kurgan, three of the eight species were infected (37.5%). In Tyumen, two of the fourteen species were infected (14.3%). Seven of the fourteen species in Tobolsk were found to be infected (50.0%; Table 2). The infected species were recorded in nearly equal proportions among dominant, subdominant, rare, and isolated species. The Tobolsk sample included *Ae. cantans* (4.6%), *Ae. communis* (1.6%), *Ae. behningi* (one infected female with L3 stage larvae), and *Ae. sticticus* (0.4%). Two infected *Ae. communis* females were identified in Bobrovsky, along with one infected *Ae. excrucians* in Khanty-Mansiysk. Figure 2 shows the collection sites where mosquitoes infected with *D. repens* were found. In axenic samples without *D. repens* infection, *Ae. communis* (two populations), *Ae. cinereus*, and *Ae. excrucians* were found to feature in the eudominant index of dominance, meaning that the proportion of this species within the collection was high (ID >30%). *Ae. punctor*, *Ae. communis*, and *Ae. diantaeus* were found to be dominant (ID 15–30%) (Table S2). In samples with an infection, *Ae. flavescens*, *Ae. euedes*, and *Ae. rossicus* were noted as eudominants, while *Ae. euedes*, *Ae. cyprius*, and *Ae. excrucians* were noted as dominants (Table 2).

The populations of Kurgan, Tyumen, and Tobolsk differed in composition and species dominance (Table S3). The Kurgan sample differed from the Tyumen sample by five species (Jacquard species similarity index, IL = 50.0%), from the Tobolsk sample by seven species (IL = 31.3%), and from both by four species (IL = 52.9%; see Table 2 and Table S4). Tyumen occupied an intermediate position between Kurgan and Tobolsk, corresponding to the latitudinal gradient of SET accumulation (Table 1, Figure 1 and Figure 2). The proportion of potential transmitters in Kurgan (92.5 ± 1.3%) was higher than in Tyumen (80.5 ± 3.3%) and Tobolsk (77.3 ± 2.8%; *p* < 0.01 in both cases). The comparative results of the study of potential vectors of *Dirofilaria* spp. in Western Siberia and the European part of the Russian Federation are summarized in Table 3.

**Figure 5:**
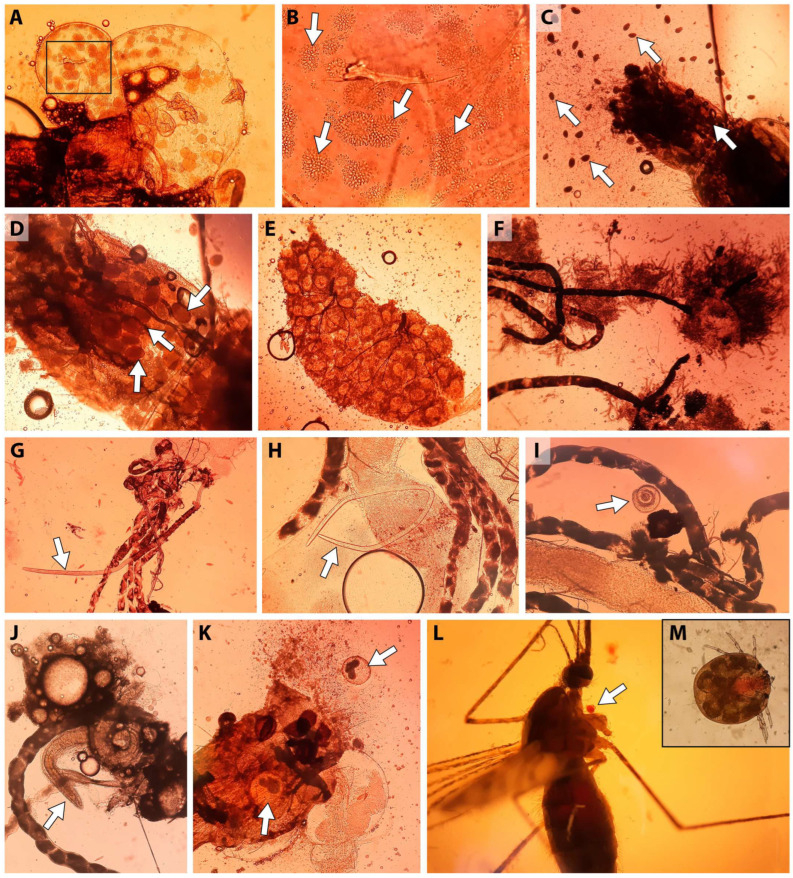
Examples of infections, other than *Dirofilaria repens*, determined in dissected adult mosquitoes. (**A**) An infection of the posterior intestine of *Aedes* with ascogregarins. (**B**) An enlarged view of the area indicated by the rectangle in panel (**A**). The infection with ascogregarins is shown by arrows. (**C**) A female *Ae. communis* from Talinka on 18 June 2021 with ciliate infestation. The infection is shown by arrows. (**D**) The same image enlarged. The infection is shown by arrows. (**E**) Infection of the ovary, presumably mycosis. (**F**) Mycosis in a female *Ae. flavescens* in Kurgan on 22 June 2021. (**G**) A female *Ae. cyprius* with an infection involving a partially melanized nematode in Kurgan on 22 June 2021. (**H**) A female *Ae. cyprius* with a nematode infection, shown by arrow, in Kurgan on 22 June 2021. (**I**) A female *Ae. euedes* with a nematode infection, shown by arrow, in Tyumen, Olovyannikov Pond on 24 June 2021. (**J**) Female *Ae. euedes* with nematode infestation shown by arrow, in Tyumen, Olovyannikov Pond on 24 June 2021. (**K**) A female *Ae. excrucians* infected with trematodes, shown by arrow, in Tobolsk on 21 June 2021. (**L**) A female *An. messeae* s.l. infected with a water mite, shown by arrow. (**M**) A magnified image of a water mite shown in panel (**L**).

In addition to *D. repens* and *Setaria* spp., >30 cases of female *Aedes* mosquitoes were infected with non-filarial nematodes (Figure 5G–J), metacercariae (Trematoda, Figure 5K), ascogregarines (Apicomplexa), infusoria (Ciliophora, Figure 5C,D), micromycetes (Figure 5E,F), and water mites (Acariformes, Hydrachnidia, Figure 5L,M) were detected. No species parasite diagnosis was performed. Three cases of infection with non-filarial nematodes were recorded: one in *Ae. euedes* from Tyumen (EI species 3.8 ± 3.7%, EI population 0.7 ± 0.7%), and two in *Ae. cyprius* from Kurgan (EI species 1.6 ± 1.1%, EI population 0.5 ± 0.4%). Metacercariae were detected in four species of *Aedes* mosquitoes (Table S4): *Ae. euedes*, *Ae. cyprius*, *Ae. flavescens* in Kurgan; and *Ae. excrucians* in Tyumen and Tobolsk. Although the same mosquito species were affected by helminths from different groups, no cases of mixed infection were found. In addition to helminthiasis, three cases of mycoses were identified in *Ae. flavescens* from the Kurgan. Infestation with ciliates was observed in isolated northern populations: *Ae. communis*; *Ae. pionips* Dyar, 1919; and *Ae. punctor* in the Talinka pine forest and *Ae. rossicus* in the Tobolsk mixed forest.

Infections of Hydrachnidia in non-malarial mosquitoes were rare (EI—from 1.0% to 3.4%; EI—1–3 mites per individual): in Shapsha—in *Ae. euedes* and *Ae. excrucians* (1.7 ± 1.2%); in Maslov—in *Ae. excrucians* (2.0 ± 2.0%); in Tobolsk—in *Cs. morsitans*, *Ae. cinereus*, and two specimens of *Ae. cantans* (1.8 ± 0.9%); in Tyumen—in *Ae. flavescens*, *Ae. excrucians*, *Ae. communis*, and two specimens of *Ae. cantans* (3.4 ± 1.5%), as well as two females of *An. messeae* and four of *An. daciae* (EI = 75%, EI—4–10 copies/individual); and in Kurgan—in two females, *Ae. flavescens*; *Ae.* sp., *Cx. modestus* Ficalbi, 1889 (1.0 ± 0.5%). There were no patterns of distribution of Hydrachnidia by species of non-malarial mosquitoes in the habitats, and no mixtures with helminthiasis were detected. In the region, mites were more often associated with the species of the *annulipes* group: *Ae. cantans* (5.9 ± 2.9%), *Ae. excrucians* (1.9 ± 1.1%) and *Ae. flavescens* (1.4 ± 0.8%). Malaria mosquitoes proved preferable for mites in both the extent and intensity of infection (EI and II).

## 4. Discussion

In this study, we identified nine mosquito species associated with *D. repens* infection between the 55th and 61st parallels in the Irtysh Basin of Western Siberia. Seven of these species were infected with *D. repens*: *Ae. rossicus*, *Ae. cantans*, *Ae. cyprius*, *Ae. euedes*, *Ae. excrucians*, *Ae. flavescens*, and *Ae. sticticus*. However, the infective stage of the L3 parasite, which can be further transmitted to the vertebrate host, was present only in two species of *Ae. behningi* and *Ae. communis*. Previous studies (Table 3) have demonstrated the vector competence of *Ae. cantans*, *Ae. excrucians*, and *Ae. sticticus* in Europe and North America [17,40]. In this study, we revealed a *D. repens* infection in *Ae. cyprius* for the first time. Since infected *Ae. rossicus* and *Ae. euedes* have only been observed in Western Siberia [18], their association with *Dirofilaria* spp. (*D. repens* and *D. immitis*) also remained unknown before this study. A comparison with similar results in the Tomsk region reveals high similarity in mosquito species infection rates. Of the 12 species identified in the taiga zone of the Ob and Irtysh Rivers, six were found to be infected with *Dirofilaria* spp.: *Ae. rossicus*, *Ae. behningi*, *Ae. cantans*, *Ae. communis*, *Ae. euedes*, and *Ae. excrucians* (IL = 50.0%). Despite being assessed as extremely rare in Tomsk, the presence of the three species *Ae. communis*, *Ae. rossicus*, and *Ae. euedes* in the Irtysh Basin was not accidental [18]. In the northern forest–steppe of the Kurgan region in southern Ural, two new potential *Aedes Dirofilaria* spp. vectors have been identified in our study: *Ae. flavescens* and *Ae. cyprius*. The former has also been noted in the vicinity of Omsk [19], while the latter has not been documented in the literature. Three species of mosquitoes—*Ae. diantaeus*, *Ae. punctor*, and *Cq. richiardii* (Ficalbi, 1889)—were not associated with *Dirofilaria* spp. infection in the Irtysh region. The incidence of *Ae. punctor* in the Tomsk region was less than 1%, which required a large sample size to detect it. The association of *Ae. diantaeus* and *Cq. richiardii* with *Dirofilaria* spp. infection was more pronounced in July and August rather than in the earlier summer months. This is likely explained by the high proportion of autogenic first-generation individuals for *Cq. richiardii* [41]. The appearance of *Ae. sticticus* in Tobolsk, which is known for its vector abilities in Europe and North America [17,40,42,43,44], was unusual. This species is classified as flood-like, with cyclical outbreaks of abundance [45]. During these outbreaks, the vector properties of the populations apparently increase.

Twenty-one species of mosquitoes associated with *Dirofilaria* spp. have been found in the European part of Russia, and 15 of these species have been demonstrated to be vector competent to *Dirofilaria* spp. [14,15,17,46]. These studies took place much farther south (43–56° N) than the surveyed territory in this study (55–67° N; Table 1), which explains the difference in species composition. Of the 45 *Dirofilaria* spp. vector species identified in natural populations in Europe [13] and Western Siberia [18,19,20], 14 (31.1%) were absent from our study. These 14 species include *Cx. theileri*, *Ae. albopictus*, *Ae. aegypti*, *Ae. koreicus*, *An. plumbeus*, *An. hyrcanus*, *An. atroparvus*, *An. maculipennis* s.s., *An. sacharovi*, *An. algeriensis*, *An. pseudopictus*, *Cs. annulata*, and *Uranotaenia unguiculata*, because they do not occur in this region. Another 11 species (26.7%), including *Ae. intrudens* (Dyar 1919), *Ae. cataphylla* (Dyar 1916), *Ae. riparius*, *Ae. leucomelas*, *Ae. cataphylla*, *Cs. alaskaensis*, and *Cs. longiareolata*, were present in Western Siberia but do not exhibit vector activity. One possible reason is the replacement of a highly competent subspecies with a less competent one [33]. For instance, the active European vector *Ae. vexans* ssp. *vexans* (Meigen, 1830) has been replaced by *Ae. vexans* ssp. *nipponii* (Theobald, 1907) in many Western Siberian habitats [39,47]. Similarly, *Culex pipiens* s.s. was replaced by *Culex torrentium* (Martini 1925) [48]. The natural infection rate of the autogenous form of *Cx. pipiens* var. *molestus* was significantly lower than that of the diapausing *Cx. pipiens* var. *pipiens* [49]. Such transitions complicate the identification of vectors in local habitats, yet they also present new opportunities for understanding the parasite’s adaptation strategy to new host species.

Distinctions in the abilities of different mosquito species to carry *Dirofilaria* spp. parasites are evident in the decreasing proportion of these species in northern and eastern habitats. In this study in the Irtysh Basin of Western Siberia, it was 34.6% (9 out of 26). In contrast, in Tula, 70.6% of non-malarial mosquito species were associated with *Dirofilaria* spp. (12 out of 17 included in the analysis). In Omsk, the Nizhny Novgorod region, and Tomsk, the percentages were 80.0% (8 out of 10), 58.3% (7 out of 12), and 33.0% (9 out of 27), respectively [14,15,18,19]. The number of mosquito species increases in forest areas, while the proportion of species associated with *Dirofilaria* spp. decreases. This may be due to the specific blood-feeding behaviors of the mosquitoes [50] and the extent to which their definitive hosts are infected and their abundance. In Siberian dog populations, only about 3% of dogs were infected [25]. Fifteen species of genus *Aedes* were found to be infected with *Dirofilaria* spp. in Western Siberia, except for *Ae. rossicus*, *Ae. cinereus* and *Ae. vexans*, which belong to the *Ochlerotatus* subgenus. In the taiga, the primary agent of transmission shifts from *Ae. cinereus* to *Ae. rossicus* [14,18,19]. However, the latter species is often inferior to the former in terms of numbers and eurytopicity. The reason for this substitution is unclear, but their identification may provide a key to understanding the processes occurring during the expansion of *Dirofilaria* spp. into Western Siberia. The situation is also intriguing because determining the species of the *Aedes* subgenus is difficult, as it may range from two to six [9] due to shifting range boundaries during climate warming. Another subgenus of the genus *Aedes* is *Stegomia*, which has enhanced vector properties. Only one species from this subgenus, *Ae. sibiricus* [51], has been found in Western Siberia [52]. This species is more characteristic of Eastern Siberia and Southern Primorye, though its vector competence remains unknown [51].

Our study recorded vector-competent females of *Aedes* mosquitoes up to 60° N in Bobrovsky (Figure 1, Table 1), one degree north of the previous record for *An. beklemishevi* in the Tomsk region [20]. The difficulty of detecting infected females is related to the discreet nature of the parasite’s maturation process, which depends on the distribution of effective temperatures throughout the season [29,30], as well as the abundance of vectors, which depends on microclimatic conditions. *Dirofilaria repens* larvae have been found in female *Anopheles* mosquitoes as far north as Priobye village in the Khanty-Mansiysk Autonomous Okrug, which is located at 62.5° N [5]. However, the transmission efficiency and vector activity north of 60° N are currently unknown and require further attention. At present, there is no evidence suggesting that temperature restricts the transmission of the parasite within the identified infection zone. The only reason microfilaria do not mature to the infective stage is the immune properties of the mosquito species. According to the average daily external incubation model [29,30], a study of seasonal temperature dynamics in Ob–Irtysh Basin habitats shows that microfilaria can mature under current conditions up to 64° N. The average monthly temperature in July in Berezovo (16.7 °C) corresponds to the amount of heat required to develop 1.5 to 2 TC (Figure 3B). Using the hourly model [38] to recalculate the SET increases the number of TC per season by 12–25%. The clock model more accurately describes the development of microfilaria in a continental climate with significant diurnal temperature variations. Reaching the three TC thresholds indicates that the parasite is in its comfort zone with the optimal humidity [53]. Experiments and natural observations have shown that the number of DD necessary for the life cycle of ectothermal organisms decreases as they move northward into environments with higher humidity, longer photoperiods, and greater daily temperature amplitudes [40,54,55]. However, the development threshold of filariids (14 °C) does not appear to decrease under circumpolar conditions [56]. The current warming phase of the carbon cycle contributes to the northward shift in the parasite’s range boundary, worsening the epidemic forecast [1,32].

In addition to *D. repens* infection, our study revealed the presence of other parasites in *Aedes* mosquitoes in the Irtysh Basin of Western Siberia. A previous study conducted in the Tomsk region also found the presence of nematodes other than *Dirofilaria* spp. in mosquitoes of the genus *Aedes*. [18]. Five species of mosquitoes were identified as infected here: *Ae. punctor*, *Ae. cantans*, *Ae. euedes*, *Ae. excrucians*, and *Ae. behningi*. In the B. Sarovka and Molchanovo populations in the Tomsk region, three species were found to be infected with filariids of the *intrudens* group: *Ae. diantaeus*, *Ae. intrudens*, and *Ae. pullatus* (Burlak, unpublished data). The mermitid species found in *Ae. cantans* has been reported as a representative of *Culicimermis schakhovii* [57]. *Aedes euedes* was previously identified as a vector of Omsk hemorrhagic fever [47], but it was not known to carry *Dirofilaria* spp. outside of Western Siberia. However, *Ae. intrudens* has been mentioned as a carrier of *Dirofilaria* spp. in the European part of Russia and *Ae. pullatus* has also been considered a potential carrier [14].

Trematodosis (AI = 1–7) caused by metacercariae (Trematoda) was found in three habitats of the Irtysh Basin (Kurgan, Tyumen, Tobolsk). Six infected individuals (0.8%) of four species were found (*Ae. flavescens*, *Ae. excrucians*, *Ae. euedes*, and *Ae. cyprius*; Table S4). The discovery of trematodes is quite unusual: no metacercariae were found in non-malarial mosquitoes in the Tomsk region (Burlak, unpublished data). Based on our observations, we hypothesize that helminthic infestations increase the likelihood that certain mosquito species will transmit nematodes, including *Dirofilaria* spp. The prolonged interaction between mosquitoes and helminths creates pathways that allow parasites to evade the host’s immune system. Therefore, the presence of setariasis or mermitid infestation creates conditions that allow the development of vector properties for *Dirofilaria* spp. infection. Some species of the genus *Aedes* exhibit a high capacity for melanization. For instance, *Ae. diantaeus* melanizes microfilariae closer to the end of L2, unlike *Anopheles* or other *Aedes* species, which melanize microfilariae at the L1 stage [20,58]. This suggests a delayed inducible reactivity in forest species. Some other species of the genus *Aedes* typically exhibit similar responses [59].

Table 3 summarizes data from the past decade of studies on *Dirofilaria* spp. in mosquito populations in the European part of Russia and Western Siberia. These data enabled us to identify the range of mosquito species associated with *Dirofilaria* spp. However, these data require additional investigation on changes in the species composition of vectors during the transition from the forest-steppe to the taiga zone and on the effectiveness of vector species. These studies will significantly improve the accuracy of epidemic forecasts and contribute to our understanding of helminth–host interactions during the spread of invasive species.

## 5. Conclusions

In this study, it was determined that *D. repens* is widespread in the Irtysh region, ranging from Kurgan to Khanty-Mansiysk. Infections with this species were found in populations of *Aedes* mosquitoes up to the latitude of Khanty-Mansiysk (61st parallel). Transmission of the parasite by local species was detected up to the 60th parallel (Bobrovskoye), indicating the parasite’s successful adaptation to high latitudes. Nine species of *Aedes* mosquitoes were infected with *D. repens* were found: *Ae. rossicus*, *Ae. behningi*, *Ae. cantans*, *Ae. communis*, *Ae. cyprius*, *Ae. euedes*, *Ae. excrucians*, *Ae. flavescens*, and *Ae. sticticus*. Two species, *Ae. behningi* and *Ae. communis*, appeared to be vector-competent to *D. repens* in the taiga zone. This study demonstrated that mosquito species infected with *D. repens* across different locations in the taiga zone were highly similar to one another, yet remarkably different from those in the forest–steppe zone. Mosquito species infected with *D. repens* were also capable of carrying other local helminths, including filarial worms, nematodes, and trematodes. The presence of these parasites likely enhanced the mosquito’s ability to carry and transmit *D. repens*. Analysis of external incubation temperatures (EITs) across various mosquito habitats revealed that the development duration of the *D. repens* daily infection cohort increased by five days per latitude degree.

## Figures and Tables

**Table 1:** *Dirofilaria repens* and other infections in mosquitoes from the Ob–Irtysh Basin.

	Location	Date	Time	TC	N/n	Extent of Infection, EI, %			
						*Dirofilaria repens*	Other Nematoda spp.	Trematoda spp.	Hydrachnidia spp.
1	Salekhard **	9 August 2020	18	0.74	28/0	0	3.6 ± 3.5 #	0	0
2	Berezovo **	29 June 2021	19	0.31	193/0	0	0	0	0
3	Peregrobnoe *	26 June 2021	18	0.61	72/0	0	0	0	0
4	Sergino	18 June 2021	13	0.61	132/0	0	0	0	0
5	Talinka	18 June 2021	16	0.61	279/0	0	0	0	0
6	Shapsha	19 June 2021	10	0.71	123/0	0	0	0	1.6 ± 1.1
7	Khanty-Mansiysk 1 **	7 August 2020	19	2.54	54/0	0	0	0	0
8	Khanty-Mansiysk 2 **	28 June 2021	8	0.79	233/1	0.4 ± 0.4	0	0	0
9	Bobrovsky *	25 June 2021	20	1.00	98/2	2.0 ± 1.4	0	0	0
10	Tobolsk 1	21 June 2021	16	1.12	65/8	12.3 ± 4.1	0	1.5 ± 1.5	4.6 ± 2.6
11	Tobolsk 2	25 June 2021	11	1.14	160/9	5.6 ± 1.8	0	0	0.6 ± 0.6
12	Tobolsk 3 **	18 September 2021	-	3.75	10/0	0	0	0	0
13	Maslova	21 June 2021	10	1.12	50/0	0	0	0	2.0 ± 2.0
14	Tyumen 1	23 June 2021	21	1.58	35/0	0	0	0	2.9 ± 2.9
15	Tyumen 2	24 June 2021	6	1.58	75/1	1.3 ± 1.3	1.3 ± 1.3	1.3 ± 1.3	4.0 ± 2.3
16	Tyumen 3	24 June 2021	15	1.58	38/1	2.6 ± 2.6 &	0	0	18.4 ± 6.3
17	Bolshiye Akiyary	24 June 2021	13	1.58	173/0	0	0	0	0
18	Kurgan	22 June 2021	18	2.06	387/3	0.8 ± 0.5	0.5 ± 0.4	1.0 ± 0.5	1.3 ± 0.6
	Total				2205/25	1.1 ± 0.2	0.2 ± 0.1	0.3 ± 0.1	1.0 ± 0.2

Note: TC indicates the number of transmission cycles at the time of sampling (the sum of effective temperatures divided by 130). N is the sample size, and n is the number of infected females. An asterisk (*) represents habitats in which the species composition of mosquitoes was not determined. A double asterisk (**) represents habitats in which mosquito infestation was determined only by PCR. In Khanty-Mansiysk, 49 of 233 females were dissected without determining the mosquito species. The infection status of the remaining 184 females was assessed by PCR following species identification. Tyumen 2 and 3 samples were collected from the same place but at different times. The # symbol represents *Setaria* sp. The & symbol represents *Dirofilaria* sp.

**Table 2:** The *Dirofilaria repens* infection found in mosquitoes in the Irtysh Basin.

	Species		Khanty-Mansiysk	Tobolsk	Tyumen	Kurgan	Total
1	*Ae. rossicus*	N/n	0	120/5	3	0	123/5
		ID		53.3 ± 3.3	2.0 ± 1.2		13.0 ± 1.1
		EI		4.2 ± 1.8			4.1 ± 1.8
2	*Ae. behningi*	N/n	0	1/1	1	0	2/1
		ID		0.4 ± 0.4	0.7 ± 0.7		0.2 ± 0.1
		EI		100	0		50.0 ± 35.4
3	*Ae. cantans*	N/n	1	26/4	8	1	36/4
		ID	0.5 ± 0.5	11.6 ± 2.1	5.4 ± 1.9	0.3 ± 0.3	3.8 ± 0.6
		EI	0	15.4 ± 7.1	0	0	11.1 ± 5.2
4	*Ae. euedes*	N/n	0	5/2	26/1	127/1	158/2
		ID		2.2	17.6	32.9	16.2
		EI		9.1 ± 6.1	3.8 ± 3.8	0.8 ± 0.8	1.3 ± 0.9
5	*Ae. cyprius*	N/n	0		2	60/1	62/1
		ID			1.4	15.5	6.6 ± 0.8
		EI			0	1.7 ± 1.7	1.6 ± 1.6
6	*Ae. excrucians*	N/n	160/1	11/1	32/1	2	205/3
		ID	87.0 ± 2.5	4.9 ± 1.4	21.6 ± 3.4	0.5 ± 0.4	21.7 ± 1.3
		EI	0.6 ± 0.6	9.1 ± 8.7	3.1 ± 3.1	-	1.5 ± 0.8
7	*Ae. flavescens*	N/n	0	0	45	167/1	212/1
		ID			30.4	43.3	22.5
		EI			0	0.6 ± 0.6	0.5 ± 0.5
8	*Ae. sp. gr. annulipes*	N/n	0	1/1	0	0	1/1
		ID		0.4			0.2
		EI		100			100
9	*Ae. communis*	N/n	1	11/2	1	0	13/2
		ID	0.5 ± 0.5	4.9 ± 1.4	0.7 ± 0.7		1.4 ± 0.4
		EI	0	18.2 ± 11.6	0		15.4 ± 10.0
10	*Ae. sticticus*	N/n	0	1/1	0	0	1/1
		ID		0.4 ± 0.4			0.2 ± 0.2
		EI		100			100
Total		N/n	184/1	225/17	148/2	386/3	943/23
		ID	19.5 ± 1.3	23.9 ± 1.4	15.7 ± 1.3	40.9 ± 1.6	100
		EI	0.5 ± 0.5	7.6 ± 1.8	1.4 ± 0.9	0.8 ± 0.4	2.4 ± 0.5

Note: N indicates individuals of the species, n indicates infected females, ID refers to the index of dominance, and EI represents the extent of infection.

**Table 3:** The distribution of vector species in the regions of the European part of Russia and Western Siberia.

Northern Latitude, Degrees	Belt *	The European Part of Russia	Western Siberia **	Western Siberia ***
63–67	Northern Taiga	No data	No data	(*Ae. riparius*)
59–63	The Middle Taiga	No data	*An. beklemishevi*, *An. daciae*, *An. messeae* [5]	*Ae. excrucians*, *Ae. communis*
57–59	Southern Taiga	No data	*An. beklemishevi*, *An. daciae*, *An. messeae* [5,20], *Ae. excrucians* [18], (*Ae. diantaeus*, *Ae. intrudens*, *Ae. pullatus*, Burlak, unpublished data)	*Ae. rossicus*, *Ae. behningi*, *Ae. cantans*, *Ae. communis*, *Ae. euedes*, *Ae. sticticus*, *Ae. gr. annulipes*
55.5–57	Aspen and birch forests	*An. messeae*, *Ae. cinereus*, *Ae. vexans*, *Ae. geniculatus*, *Ae. cantans*, *Ae. communis*, *Ae. intrudens*, *Cx. pipiens*, *Ae. excrucians*, *Cq. richiardii* [15].	*An. beklemishevi*, *An. daciae*, *An. messeae* [5,20], *Ae. excrucians*, *Ae. rossicus*, *Ae. behningi*, *Ae. cantans*, *Ae. communis*, *Ae. euedes*, *Ae. diantaeus*, *Ae. punctor*, *Cq. richiardii* [18]	*Ae. excrucians*, *Ae. euedes*
52–55.5	Forest steppe	*Ae. cinereus*, *Ae. vexans*, *Ae. geniculatus*, *Ae. cantans*, *Ae. communis*, *Ae. intrudens*, *Ae. cataphylla*, *Ae. punctor*, *Ae. leucomelas*, *Ae. sticticus*, *Cx. pipiens*, *Cs. alaskaensis*, *An. maculipennis* [14]	*An. beklemishevi*, *An. daciae*, *An. messeae* [5], *Ae. flavescens*, *Ae. excrucians*, *Ae. cinereus*, *Ae. vexans*, *Ae. caspius*, *Ae. dorsalis*, *Cx. modestus*, *Cx. pipiens* [19]	*Ae. cyprius*, *Ae. flavescens*, *Ae. euedes*
44–52	Steppe and further south	*Ae. caspius*, *Ae. dorsalis*, *Cx. pipiens*, *Cx. molestus*, *Cx. modestus*, *An. maculipennis* [2], *Ae. aegypti*, *Ae. albopictus* [17]	No data	No data

Note: Asterisk (*) stands for Western Siberia; double asterisk (**) stands for literature data; triple asterisk (***) indicates data from this article. The species infected by other filariids are indicated in parentheses.

## Data Availability

All data are available throughout the text and Supplementary Materials. Terrestrial ecoregions of the world shown in Figure 1 are available from [60].

## Supplementary Materials

The following supporting information can be downloaded at: https://www.mdpi.com/article/10.3390/insects17040398/s1. Figure S1: Mosquito collection sites. Figure S2: Shapsha, Shaitanskaya River floodplain, 3 August 2020. Bolshye Akiyary—shore of Lake Inyk, 24 June 2021. Berezovo—roadside moat, 29 June 2021. Talinka, a body of water with no name, 4 August 2020. Kurgan, forest steppe, 22 June 2021. Maslova, osinovy kolok, bank of the Zaimskaya River, 21 June 2021. Salekhard, Ob Riverbank (ferry crossing), 9 August 2020. Sergino, a stream without a name, 18 June 2021; Table S1: The characteristics of the biotopes in the mosquito collection sites in the Ob–Irtysh Basin; Table S2: The species composition of mosquitoes in the Ob–Irtysh Basin habitats; Table S3: The reliability of differences in dominance indices among mosquito species infected with *Dirofilaria repens*; Table S4: Trematode and metacercariae infections in mosquito species.

## Abbreviations

The following abbreviations are used in this manuscript:

PCR	Polymerase chain reaction.
COI	Cytochrome oxidase subunit I.
ITS2	Internal transcribed spacer 2.
RFLP	Restriction fragment length polymorphism.
TC	Transmission cycle.
SET	Sum of the daily average temperature.
DD	Units of *Dirofilaria* spp. development.
EIT	Incubation temperature.
EI	Extensiveness of infection.
IL	Jacquard species similarity index.
II	Intensity of infection.
ID	Index of dominance.
MIR	Minimum infection rate.
