# Supplementary material for: The Expansion of Dirofilaria repens in the Irtysh Basin of Western Siberia Is Associated with Nine Species of Aedes Mosquitoes
Source: Insects. 2026 Apr 7;17(4):398. doi: 10.3390/insects17040398 (PMC13115762; doi:10.3390/insects17040398)
Supplement: Supplementary file 1 [file insects-17-00398-s001.zip › Figure_S2.pdf]

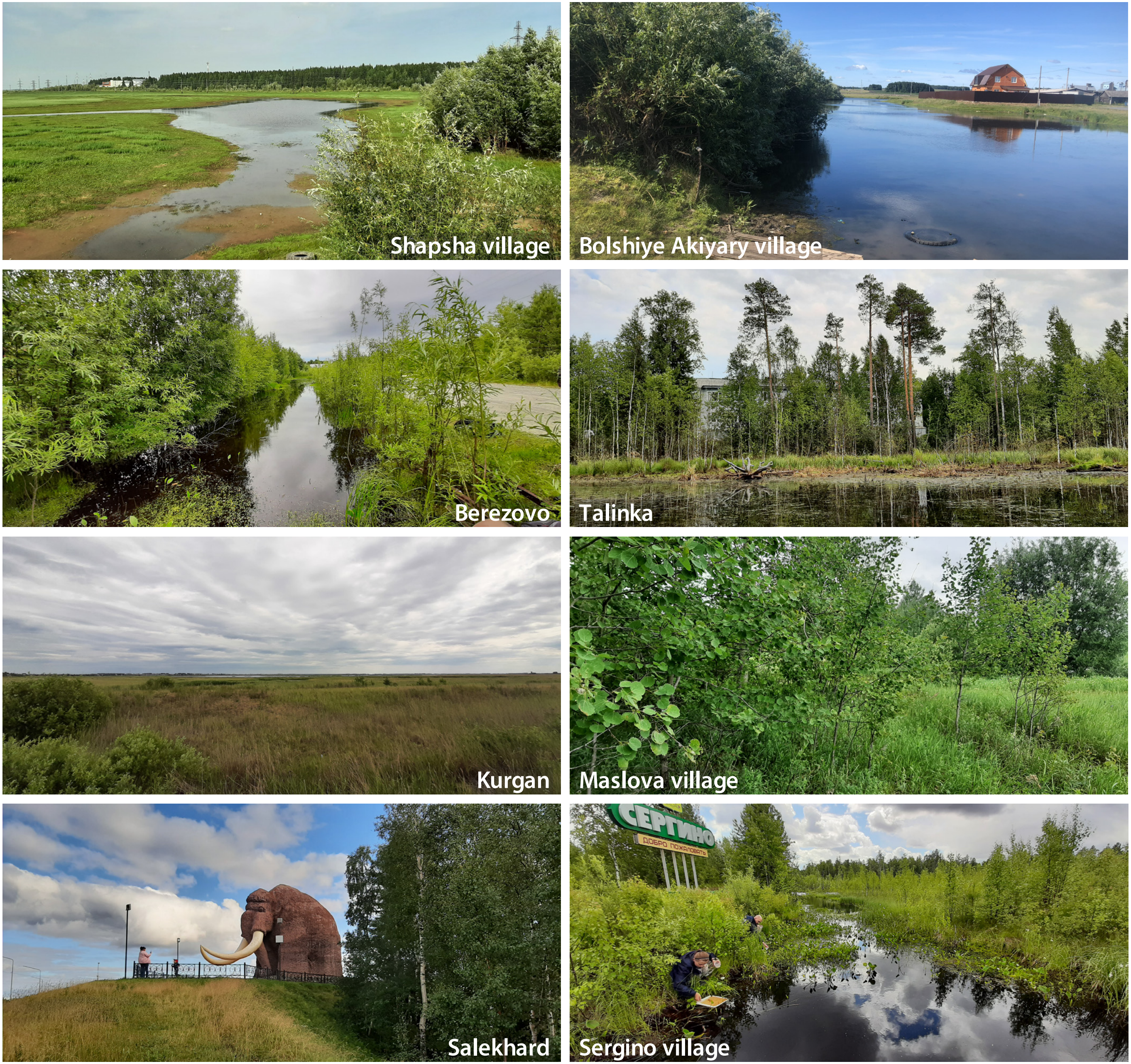
Shapsha village
Bolshiye Akiyary village
Berezovo
Talinka
Kurgan
Maslova village
Salekhard
СЕРГИНО
Sergino village
