# Supplementary material for: The Expansion of Dirofilaria repens in the Irtysh Basin of Western Siberia Is Associated with Nine Species of Aedes Mosquitoes
Source: Insects. 2026 Apr 7;17(4):398. doi: 10.3390/insects17040398 (PMC13115762; doi:10.3390/insects17040398)
Supplement: Supplementary file 1 [file insects-17-00398-s001.zip › STables.pdf]

Table S1. Characteristics of the biotopes in the mosquito collection sites in the Ob-Irtysh basin

№	Collection site	Region	Latitude	Longitude	River	Tributary	Object	Vegetation
1	Salekhard	Yamal	66°37'23"	66°33'36"	Ob	-	Mammoth Monument / Deciduous spike / swamp	birch tree / *Betula sp.*
2	Berezovo	Yugra	63°56'41"	65°01'18	Ob	Golchenlor River	Mixed forest / upland	Fir, pine, lingonberry, blueberry, rosemary *Abies sibirica, Pinus silvestris, Vaccinium vitis-idaea, V. mirtillus, Ledum palustre*
3	Peregrobnoe village	Yugra	62°58'14"	65°04'17"	Ob	-	Mixed forest / upland	ash, birch, aspen *Sorbus aucuparia, Betula sp., Populus tremula*
4	Sergino village	Yugra	62°28'13"	65°34'20"	Ob	Karemposl Bayou	The shore of the swamp / stella	Mixed forest
5	Talinka	Yugra	61°33'23"	66°26'45"	Ob	Endyr River	Pine forest	Pinus silvestris, Sorbus aucuparia
6	Shapsha village	Yugra	61°05'14"	69°27'43"	Ob	Shaitan River	The edge of the coniferous forest / swamp	Coniferous forest, birch, aspen, chin, peas, horsetail, lapchatka *Lathyrus sp., Vicia sp., Equisetum sp., Potentilla sp.*
7	Khanty-Mansiysk	Yugra	60°59'49"	69°01'02"	Ob	Irtysh	Samarovsky Chugas Park / wooden bridges	Dark coniferous forest
8	Bobrovsky village	Yugra	59°58'28"	69°55'45"	Irtysh	Bobrovka River	The edge of the coniferous forest / swamp	Coniferous forest, sedges, cereals
9	Tobolsk	Tyumen	58°12'15"	68°14'48"	Irtysh	Tobol	Irtysh floodplain / aspen-birch forest	Aspen, birch, willow, horsetails, legumes
10	Maslova village	Tyumen	58°04'57"	68°25'01"	Irtysh	Zaimskaya River	Floodplain of the Zaimskaya River / deciduous forest / cemetery	Aspen spike
11	Tyumen 1	Tyumen	57°10'01"	65°37'16"	Tura	-	Gilevsky Park / Floodplain Tours	Willow, islands of various grasses
12	Tyumen 2	Tyumen	57°10'04"	65°26'50"	Tura	Olovyannikov Pond	Zatyumensky Ecopark / Olovyannikov Pond	Mixed forest, mixed grasses
13	Bolshiye Akiyary village	Tyumen	56°57'27"	65°22'06"	Pyshma	Inyk Lake	Inyk Lake floodplain	Mixed forest, sedges, mixed grasses
14	Kurgan	Kurgan	55°25'38"	65°19'51.5"	Tobol	Bitevka River	Central Recreation Park / city center / Bitevka River	Aspen, birch, willow, grass, bonfire (*Bromus sp.*)

Table S3. The reliability of differences in dominance indices among mosquito species infected with dirofilariae.

№	Species	Comparison option (p <)		
		Kurgan/Tyumen	Kurgan/Tobolsk	Tyumen/Tobolsk
1	*Ae. flavescens*	0.05	0.01	0.01
2	*Ae. euedes*	0.01	0.01	0.01
3	*Ae. cyprius*	0.01	0.01	—
4	*Ae. excrucians*	0.01	0.05	0.01
5	*Ae. cantans*	0.05	0.01	—
6	*Ae. communis*	—	0.01	—
7	*Ae. rossicus*	—	0.01	0.01
8	*Ae. behningi*	—	—	—
9	*Ae. sticticus*	—	—	—

Table S4. The trematode and metacercariae infection in mosquito species.

№	Species	Tobolsk		Tyumen		Kurgan		Total	
		N/n	EI	N/n	EI	N/n	EI	N/n	EI
1	*Ae. excrucians*	11/1	9.1±8.7	32/1	3.1±3.1	2/0	0	45/2	4.4±3.1
2	*Ae. cyprius*	0	—	2/0	0	60/1	1.7±1.7	62/1	1.6±1.6
3	*Ae. euedes*	5/0	0	26/0	0	127/2	1.6±1.1	158/2	1.3±0.9
4	*Ae. flavescens*	0	—	45/0	0	167/1	0.6±0.6	212/1	0.5±0.5
	**Extent of invasion, %**	225/1	0.4±0.4	148/1	0.7±0.7	387/4	1.0±0.5	760/6	0.8±0.3

Note: N indicates individuals of the species, n stands for infected females. EI represents the extent of invasion.
